# Use of Raw Peach Gum as a Sustainable Additive for the Development of Water-Sensitive and Biodegradable Thermoplastic Starch Films

**DOI:** 10.3390/polym15163359

**Published:** 2023-08-10

**Authors:** Andrea Juan-Polo, Cristina Pavon, Harrison de la Rosa-Ramírez, Juan López-Martínez

**Affiliations:** Instituto de Tecnología de Materiales, Universitat Politècnica de València (UPV), 03801 Alcoy, Alicante, Spain; anjuapo@epsa.upv.es (A.J.-P.); hardela@epsa.upv.es (H.d.l.R.-R.); jlopezm@mcm.upv.es (J.L.-M.)

**Keywords:** thermoplastic starch, peach gum, sustainable additive, disintegration, water sensitivity

## Abstract

In this study, formulations of thermoplastic starch (TPS) with 5, 10, and 15 parts per hundred resin (phr) of raw peach gum (PG) were prepared by melt extrusion followed by injection molding to obtain standard specimens for characterization. In addition, biodegradable films were developed by compression molding. It was determined that TPS with 5 phr and 10 phr of PG presented similar mechanical behavior to pure TPS after the processing. However, results indicated that adding PG in 10 phr slowed down the starch’s retrogradation, delaying the TPS structure’s stiffening. Moreover, the TPS–PG formulations presented improved solubility, which increased by 24% with 10 and 15 phr of PG compared to that shown for TPS. Additionally, PG enhanced the compostability of TPS, causing the sample to disintegrate in a shorter period. In conclusion, it was determined that raw PG added in 10 phr could be added as a sustainable additive to modify the biodegradation and water sensitivity of TPS without affecting its mechanical behavior after processing and delaying the retrogradation of the TPS structure, increasing its shelf life.

## 1. Introduction

Bioplastics can be produced from biomass, are susceptible to biodegradation, or meet both conditions. Due to their low environmental impact, the most interesting bioplastics come from natural sources and can biodegrade. Natural polymers of practical interest are limited to a few polysaccharides and proteins, polysaccharides being the most important. Polysaccharides account for 75% of the annual biomass production (about 170 billion tons) [1].

After cellulose, starch is the most available polysaccharide, and its industrial, non-food use is growing in production volume, particularly for preparing starch-based plastic. Currently, biopolymer production is around 2.41 million tons, of which 18.7% is produced from starch [2]. Starch is commonly used in elaborating biopolymer formulations because it is inexpensive, abundant in nature, comes from renewable sources, and has an inherent biodegradable and biocompatible nature [3,4]. However, starch in its native status does not exhibit thermoplastic behavior. Starch must be de-structured and plasticized to become a thermoplastic material [5].

Thermoplastic starch (TPS) is obtained by the action of shear forces and temperature over starch as well as the addition of a plasticizer, for instance, water, glycerol, or sorbitol [5,6]. In recent years, TPS has gained attention for developing biodegradable starch-based food-packaging materials or edible coatings [7,8]—and water-soluble films [9]. However, the industrial application of TPS is limited due to its low mechanical properties and water resistance. In addition, the changes in its structure are subject to re-crystallization and retrogradation [10,11].

Renewable polysaccharides produced from plants, such as gum rather than synthetic, are receiving more attention for their use in blends with TPS due to their biodegradability nature being environmentally friendly. For instance, adding agar in TPS to prepared products by casting or melt blending resulted in films with promising barrier and tensile properties. Moreover, the rigidity and strength significantly increased with higher agar content, while the deformability of the blends was better than that of pure TPS [12]. Chitosan was used to prepare TPS/chitosan composites and chitosan provided a reinforcing effect in TPS. Additionally, chitosan decreased the maximum water uptake of composites [13]. Cellulose was used to prepare cellulose–starch bio-composites. The results showed that the blend of starch with cellulose resulted in a not-brittle thermoplastic starch film with higher tensile strength and elongation than in unblended conditions. Furthermore, the blend of cellulose and starch also allowed for greatly reducing the mass loss caused by water immersion [14]. Starch nanocrystals (WSNC) and cellulose nanocrystals (CNC) were used to produce TPS nanocomposites, and the crystals increased the tensile strength, elongation at break, and Young’s modulus of TPS. Also, the crystals helped to reduce the oxygen permeability [15].

Peach gum (PG) is one of the world’s most abundant plant gums. Raw PG is exuded from the trunk and fruit of peach trees (*Prunus persica*) as a defense mechanism against infection, insect attack, mechanical and chemical injury, and other environmental stresses [16,17]. Raw peach gum is a solid crystalline and translucent material with brownish-yellow coloration. Raw PG contains 80–85 wt.% polysaccharide, 2–12 wt.% moisture, 0.3–4 wt.% ash, 0.2–2 wt.% protein, and traces of polyphenols and inorganic elements [17]. Raw peach gum can swell in water but has a poor water solubility [16,17]. However, the polysaccharide in PG has excellent water solubility; good biocompatibility; acceptable antioxidant, antibacterial, and film-forming properties; and has shown good application prospects in the food field, such as in microcapsule carriers, thickeners, emulsifiers, stabilizers, and candy coatings [17]. Peach gum polysaccharides (PGPs) can easily be made through the hydrolysis of raw peach gum (PG). Chemically, a PGP is an acidic heteropolysaccharide that possesses a relatively high molecular weight (>106 Da) with a branched macromolecular structure composed of (1→3)-linked β-D-Galp units in the main-chain and arabinogalactan in the side chains [16,18]. The polysaccharides in peach gum are acidic arabinogalactans with high contents of arabinose and galactose, and peach gum also has xylose, glucuronic acids, and small contents of rhamnose and mannose [19]. Arabinogalactans are water-soluble polysaccharides found in the composition of exudate resins from trees, as well as in plants, fungi, and bacteria [20]. The gums from these resins are also water-soluble; for instance, puka gum obtained from the *Meryta sinclarii* tree reaches a solubility of 25 wt.% [21], Arabic gum derived from the stems and branches of *Acacia senegal* has a solubility of 77 wt.%, and cherry gum presents a solubility of 62 wt.% [22].

**Figure 1:**
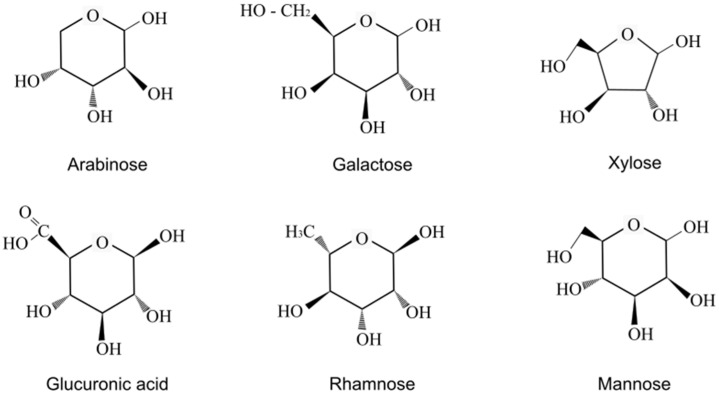
Chemical structure of the monosaccharides present in raw peach gum (PG).

The chemical structures of the monosaccharides present in peach gum are shown in Figure 1.

Peach gum is a biopolymer that is edible, non-toxic, biocompatible, digestible, renewable, and biodegradable [17,23]. Raw peach gum has been used in traditional Chinese medicine to treat hemolysis, dysentery, and diabetes, and as a pharmaceutical excipient. However, due to PG’s poor water solubility, its application is limited [17]. Nonetheless, PG has a broad potential to be used to manufacture environmentally friendly materials. Moreover, only a tiny fraction of PG has been used, and a large amount of peach gum is not utilized, which ends as waste. Furthermore, it is crucial to promote the use of natural bio-based polymers that are safer for human health and would reduce plastic waste.

Several studies have been conducted on possible uses of PG. For instance, Ref. [24] investigated the potential use of PG as a natural adsorbent for removing dyes from aqueous solutions and found that PG had excellent adsorption capabilities and high selectivity for cationic dyes. Ref. [25] studied PG polysaccharides to produce edible coatings on cherry tomatoes because of their antioxidant and antibacterial activity. The results showed that PG polysaccharides extended the shelf life of cherry tomatoes. Ref. [26] used peach gum and polyethylene oxide as fiber-forming solutions to entrap *Litsea cubeba* essential oil (EOLC). The study found that the fibers could control the release of EOLC and maintained the pH, TVB—N, and TBARS values from deterioration in the chicken. The fiber also inhibited the reproduction of *S. aureus* and *E. coli* during the storage period.

The present work aims to determine the effect of using raw peach gum as a sustainable additive in thermoplastic starch films to tune its water solubility and compostability without affecting its mechanical properties. This is the first time raw peach gum has been used as an additive in polymeric materials. Different concentrations of raw peach gum were added by melt extrusion to TPS. The resultant materials were characterized through the performance of mechanical and morphological tests, water contact angle assessment, colorimetry, water sensitivity, infrared spectroscopy evaluation, and disintegration under composting conditions.

## 2. Materials and Methods

### 2.1. Materials

Native rice starch was supplied by Manuel Riesgo S.A. (CAS: 9005-25-8). Distilled water and glycerol were used as plasticizers. Glycerol of 99% purity was supplied by Sigma Aldrich (Schnelldorf, Germany). The raw peach gum (PG) samples were collected from *Prunus dulcis* trees. The PG sample had an irregular kernel shape. Also, it presented a variation of shades between amber and a toasted brown color. Before the analysis, the raw peach gum was ground in a mortar to obtain a powder. The raw PG used was composed of Ara, Xyl, Man, Gal, Rha, and Glucuronic acids in a 35:6:4:40:13:2 molar ratio, showing an arabinogalactan structure with the typical composition of a fruit tree’s gums [22].

### 2.2. Methods

#### 2.2.1. TPS–Peach Gum Blends

**Figure 2:**
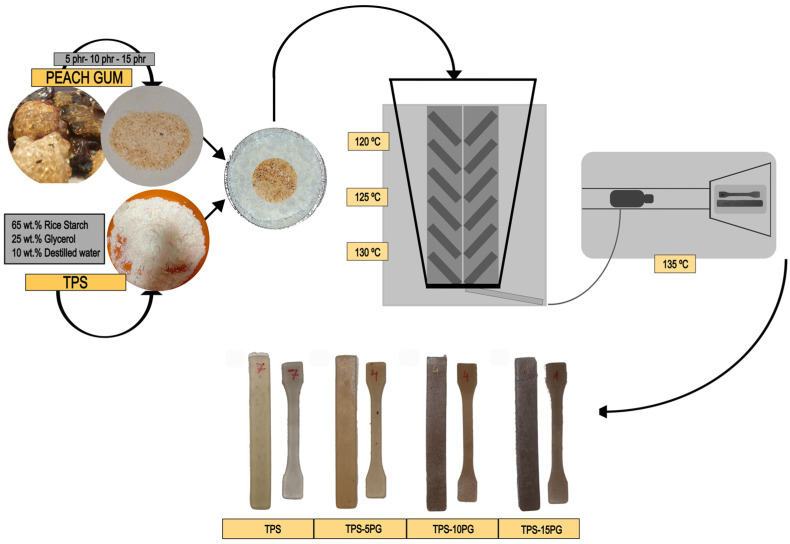
Schematic representation of TPS preparation and the processing of TPS blends with peach gum into injection-molded samples.

Figure 2 shows a schematic representation of the preparation and processing of the materials, which includes the following: (i) manual mixing, (ii) the extrusion process to obtain the TPS and PG mixtures, and (iii) injection molding to obtain mechanical characterization samples.

In brief, a mixture of 65 wt.% starch, 25 wt.% glycerol, and 10 wt.% water was manually mixed and stored in a hermetically sealed polyethylene bag for 24 h to ensure the homogeneity of the mixture and to allow the correct diffusion of the plasticizers in the starch matrix.

The mixture to obtain TPS was extruded in a micro-compounder MC 15HT Xplore with a temperature profile of 120–125–130 °C (from die to hopper) at 30 rpm for 3 min. After this time, PG was added in 5, 10, and 15 parts per hundred resin (phr). Then, the mixture was left for 3 min more. Table 1 shows the composition of the prepared samples.

The blends were further injected into Xplore’s MC 15HT micro-compounder with a mold temperature of 30 °C and injection temperature of 135 °C. The test specimens’ type 1BA (64 mm × 5 mm × 2 mm) according to ISO 527 was obtained.

#### 2.2.2. Raw Peach Gum Characterization

The morphology of raw peach gum powder was captured under an Olympus optical microscope, model SZX7 from Olympus Iberia (Barcelona, Spain), with a magnification of 1×. The image was then photographed with the help of a digital microscopic camera used in normal light mode [24,27].

#### 2.2.3. Color Characterization

The color characterization was measured using a colorimeter Colorflex-Diff2 458/08 from HunterLab (Reston, VA, USA) in the CIEL*a*b* color space. Five samples of each formulation were analyzed. The average values and the standard deviation of the coordinates L*, a*, and b* were reported together with the yellowish index (YI). The total color difference (∆E) was calculated using Equation (1) [28]:

(1) $$∆E=\sqrt{∆a^{2}+∆b^{2}+∆L^{2}}$$

Significant differences in colorimetry parameters were statistically evaluated at a 95% confidence level according to Tukey’s test using an analysis of variance (ANOVA) with OriginPro software version Origin 2018 (9.5).

#### 2.2.4. Mechanical Characterization

The mechanical characterization of the material was carried out by tensile test over the test specimens’ type 1BA prepared by injection molding. The tensile test was conducted according to ISO 527 [29]. The analysis was performed in a universal test machine Duotrac-10/1200 from Iberest (Madrid, Spain) using a 100 N load cell at a 100 mm/min crosshead rate. The tensile test was performed 1, 15, and 35 days after the specimen’s preparation to follow the mechanical properties’ evolution with time. Five samples of each formulation were analyzed in each measurement. The average values and the standard deviation of tensile strength, elongation at break, and Young’s modulus were reported. Significant differences in mechanical parameters were statistically evaluated at a 95% confidence level according to Tukey’s test using an analysis of variance (ANOVA) with OriginPro2018 software.

#### 2.2.5. Microstructural Characterization

Field Emission Scanning Microscopy (FESEM) was conducted on a Zeiss Ultra 55 microscope, Oxford Instruments (Abingdon, UK) at 1 kV. The samples were cryo-fractured before the analyses and covered with a gold–palladium alloy layer to grant electrical conduction with a Sputter Mod Coater Emitech SC7620, Quorum Technologies (East Sussex, UK).

#### 2.2.6. Water Contact Angle (WCA) Measurement

The wettability of the samples was measured with an EasyDrop-FM140 optic goniometer from Kruss Equipments (Hamburg, Germany). The obtained images were managed with Drop Shape Analysis software. Five measurements of WCA were evaluated in three specimens for each formulation. ANOVA variability analysis was conducted to determine the statistical differences between the samples with 95% confidence according to the Tukey test using OriginPro2018.

#### 2.2.7. Water Sensitivity

Water sensitivity was determined by immersing dried film pieces of TPS in 50 mL of distilled water and placing the flasks in shaking at 25 °C for 24 h [30]. The film pieces were removed (50 °C for 24 h) to determine the weight of the dry matter dispersed in water. The weight of the water-soluble matter was calculated using Equation (2) by subtracting the weight of the undissolved dry matter from the initial dry matter weight and expressed as a percentage of the initial dry matter content:

(2) $$s=\frac{w_{1}-w_{2}}{w_{1}}\cdot 100$$

where $w_{1}$ is the dry weight before the test and $w_{2}$ is the dry weight after the test. The analysis was performed over three samples of each formulation. The average value and the standard deviation were reported.

#### 2.2.8. Chemical Characterization

Attenuated total reflectance–Fourier transform infrared spectroscopy (FTIR–ATR) was applied to study the chemical interactions between TPS and PG. The analysis was performed on a Perkin Elmer Spectrum BX (FTIR system) coupled to a Pike MIRacle ATR (Beaconsfield, UK). All formulations were evaluated over a range of 4000–600 cm^−1^ with a resolution of 4 cm^−1^, a range of 2 cm^−1^, and 36 scans.

XDR was used to analyze the influence of PG on the crystallinity of thermoplastic starch. Wide-angle X-ray diffraction measurements were carried out using a Bruker D8 Advance X-ray diffractometer with a Lynxeye XE linear detector. Scattering angles (2θ) covered the ranges from 4° to 50° (θ is the Bragg angle) at a rate of 1°/min. The analyses used a 1 mm thick sample with a smooth surface.

#### 2.2.9. Disintegration under Composting Conditions

The disintegration under composting conditions test was carried out according to the parameters of the ISO-20200 standard [31] for a period of thermophilic degradation (30 days). The dry solid residue was prepared by combining 40% sawdust, 30% rabbit feed, 10% commercial compost (Mantillo, Spain), 10% corn starch, 5% sucrose, 4% corn oil, and 1% urea. Water was then added to the mixture to adjust the final water content to 55%. The wet solid residue was placed in a polypropylene container.

TPS square films of 2 cm per side with an average thickness of 1 mm were prepared for the disintegration study. The TPS–PG mixtures were compression molded at 130 °C. Film samples were dried at 50 °C for 48 h prior to testing. The samples were then weighed and placed in mesh, which allowed for access to microorganisms and moisture to facilitate their removal after treatment [32]. Samples were buried 5 cm deep in the wet solid residue of the plastic reactor and incubated under aerobic conditions (58 ± 2 °C) in an air-circulating oven. The compost was mixed gently to ensure aerobic conditions and relative humidity in the reactor, and water was added periodically [33].

The samples were taken from the container on different disintegration days (1, 4, 7, 9, 11, 14) to control the disintegration process. The collected samples were washed with distilled water, oven-dried at 50 °C for 48 h, and weighed. A visual evaluation was performed on all samples when they were removed from the composting reactor. The degree of disintegration on different days of exposure to the compost medium was calculated by normalizing the weight of the sample to the initial weight.

## 3. Results and Discussion

### 3.1. Peach Gum Characterization

**Figure 3:**
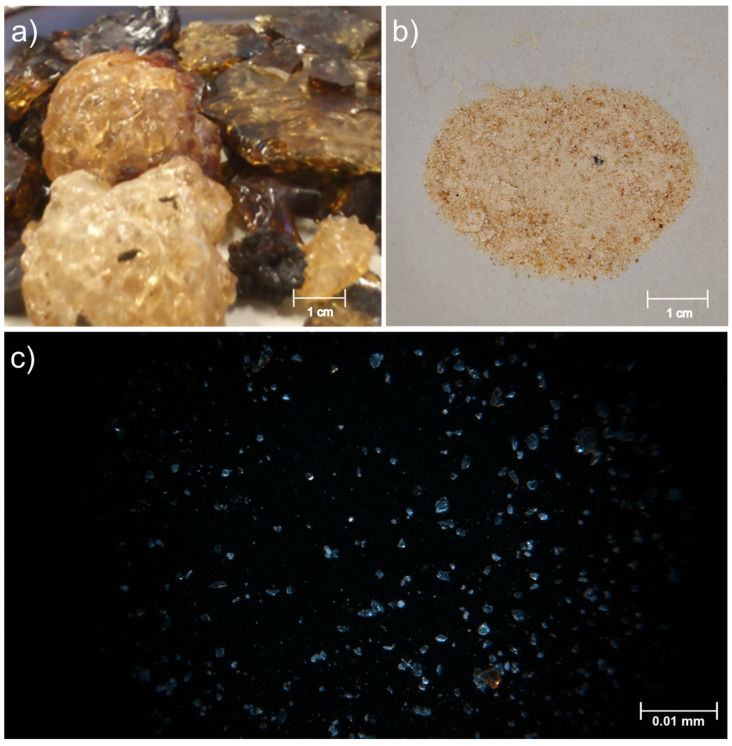
(**a**) Raw peach gum PG, (**b**) dried PG powder, and (**c**) optical microscope image of PG particles.

Figure 3 presents images of raw dried PG, PG powder, and an optical microscope image of PG particles. The photographs show that peach gum has a yellow-reddish and yellow-brownish coloration. The microscope image allowed us to determine that PG power particles were polyhedral in shape with sizes in the range of 3–15 µm in agreement with previous reports [24].

### 3.2. Color Characterization

**Figure 4:**
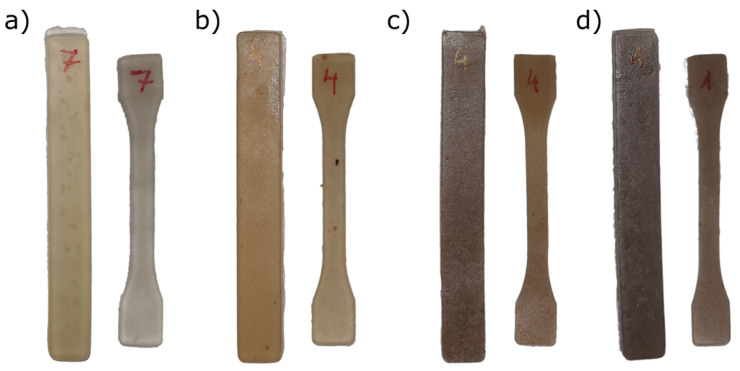
The visual appearance of TPS and TPS–PG formulations: (**a**) neat TPS, (**b**) TPS–5PG, (**c**) TPS–10PG, and (**d**) TPS–15PG.

Images of the visual appearance of TPS and TPS–PG formulations are presented in Figure 4. The color parameters in the CIEL*a*b* color space of the different formulations prepared are shown in Table 2. Figure 4 shows that all samples present a homogeneous appearance. TPS has a light coloration, whereas the addition of PG produces a darkening in the color of the samples. However, the samples’ color is uniform at a glance, indicating a good distribution of PG in TPS.

The lightness coordinates L* show that the samples are medium-light, and the TPS–PG formulation’s luminosity is statistically different (*p* < 0.05) than TPS. The a* coordinate (red–green) indicates that TPS displays a high predominance of greenish tones. When PG is added, TPS–5PG and TPS–15PG lose the hue, and the a* coordinate presents no predominant coloration as it approaches 0. However, TPS–10PG presents a reddish hue. The b* coordinate (blue–yellow) indicates that all samples have a yellow hue. However, adding PG increases the yellowish coloration significantly (*p* < 0.05), with TPS–10PG being the formulation with the highest b* value. The yellowish index (YI) increased according to the yellowish hue b* [34].

The changes observed in the CIEL*a*b* coordinates of the samples suggest that TPS’s coloration turns reddish and yellowish with the addition of PG. However, this trend stops at TPS–15PG, where it is observed that the color chordate values are like those of TPS–5PG. This result could suggest that the lack of miscibility, observed in SEM, also produces a low homogenization which influences the coloration of the sample.

The total color difference shows that adding PG affects TPS’s coloration significatively, ΔE > 2 [35], even in low contents.

### 3.3. Mechanical Characterization

**Figure 5:**
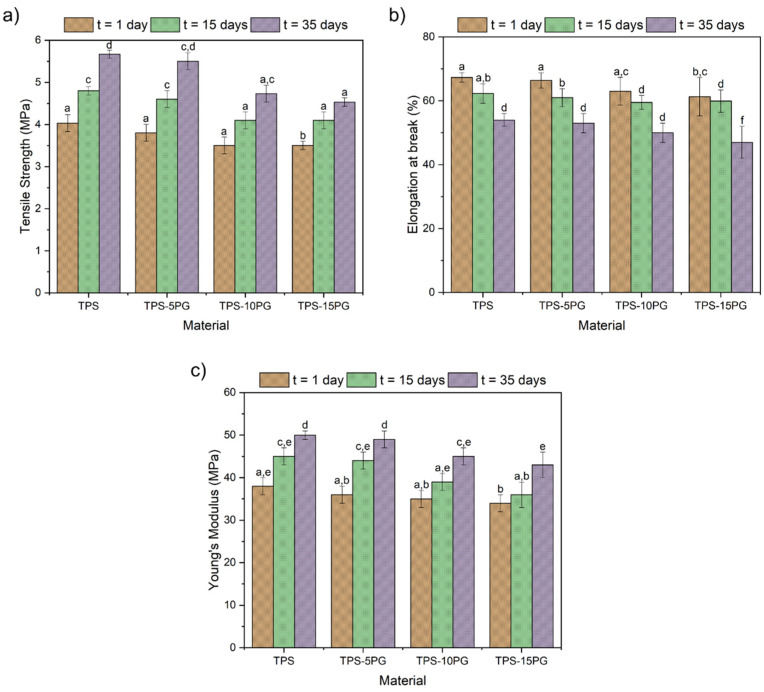
Comparison of the tensile properties of thermoplastic starch (TPS) and formulations with 5, 10, and 15 phr of peach gum (PG) with time: (**a**) tensile strength, (**b**) elongation at break, and (**c**) Young’s Modulus. ^a–f^ Different numbers show statistically significant differences between the formulations (*p* < 0.05).

Figure 5 compares the tensile properties of the TPS–PG formulations and their evolution with time. TPS presents an average tensile strength of 4.0 MPa, an elongation at break of 77.8%, and Young’s modulus of 38.8 MPa. On day 1, these parameters did not change significantly with the addition of PG in 5 and 10 phr; however, a reduction in all tensile parameters was observed in TPS–15PG.

PG is compatible with starch because of its hydroxyl groups and their similarity in structure in its polysaccharide and monosaccharides composition [36]. Therefore, 5 and 10 phr of PG blends well with thermoplastic starch. However, the monosaccharides in peach gum are not enough to affect the mechanical behavior of the TPS matrix. However, when PG is further increased to 15 phr, the miscibility limit of PG in TPS is exceeded, and PG acts as a filler whose agglomeration results in a poor stress transfer, reducing the mechanical parameter of the TPS–15PG formulation [37].

At day 15, the tensile strength of TPS increased by 18%, TPS–5PG increased by 20%, and TPS–15PG increased by 36%. In contrast, TPS–10PG did not significantly change the tensile strength. The elongation at break decreased by 7% in TPS, 9% in TPS–5PG and TPS–10PG, and 14% in TPS–15PG. On day 35, the tensile strength increased by 36% in TPS, 33% in TPS–5PG, and 43% in TPS–15PG with respect to day 1. Meanwhile, the TPS–10PG tensile strength increased by 20% with respect to day 1 but did not significatively change with respect to day 15. In addition, the elongation at break decreased in all samples between 20 to 26%. Finally, Young’s modulus gradually increased; however, TPS–10PG displayed the slowest increment in Young’s modulus compared to the other formulations.

The results show that the TPS structure progressively stiffens over time due to the recrystallization that the TPS structure is subject to because of retrogradation [38,39]. Moreover, it is seen that PG in 5 phr did not produce a significant change in the tensile properties of TPS, neither on day 1 nor over time. On the contrary, PG in 15 phr reduced the tensile properties of TPS starting with day 1. Moreover, even when PG in 10 phr did not change the TPS properties at the beginning, it seemed to delay the structure’s stiffening, as this was the formulation with the most negligible differences between its initial properties and those measured at 15 and 35 days.

### 3.4. Microstructural Characterization

**Figure 6:**
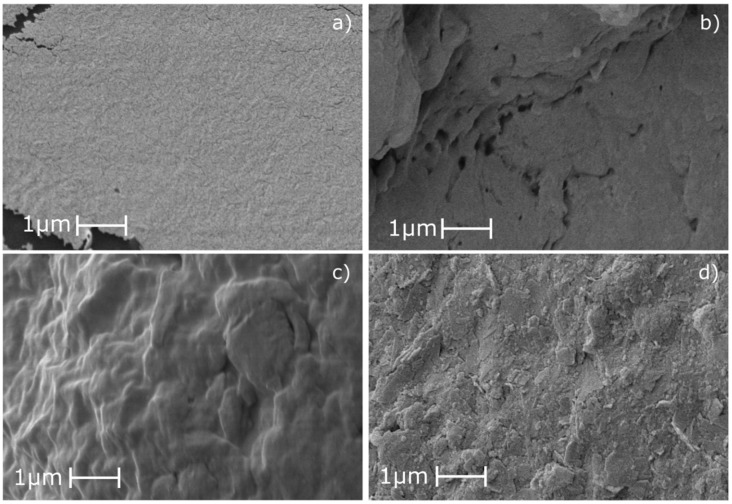
Images obtained from scanning electron microscopy analysis at 10,000×: (**a**) TPS, (**b**) TPS–5PG, (**c**) TPS–10PG, and (**d**) TPS–15PG.

Figure 6 presents the micrographs of TPS and TPS–PG formulations obtained by scanning electron microscopy. The TPS structure presented a smooth and homogeneous surface which confirms the good plasticization of starch with glycerol and water. The smooth fracture surface is typical of TPS with 25 to 30% amylose contents [11,40]. TPS added with 5 and 10 phr of PG showed no apparent phase separation, which suggests that PG was well incorporated into the TPS.

Furthermore, when PG was added in 15 phr content, TPS presented roughness over the surface, pointing to phase separation. The separation observed suggests a lack of cohesion due to low miscibility between TPS and PG at 15 phr [10]. This phase separation explains the reduction in tensile properties seen earlier in TPS–15PG. Moreover, the results indicate that PG added in 15 phr exceeds the miscibility limit between TPS and PG. This behavior has been formerly reported when using natural gums in TPS and other thermoplastic matrices [10,41,42].

### 3.5. Water Contact Angle (WCA) Measurement

**Figure 7:**
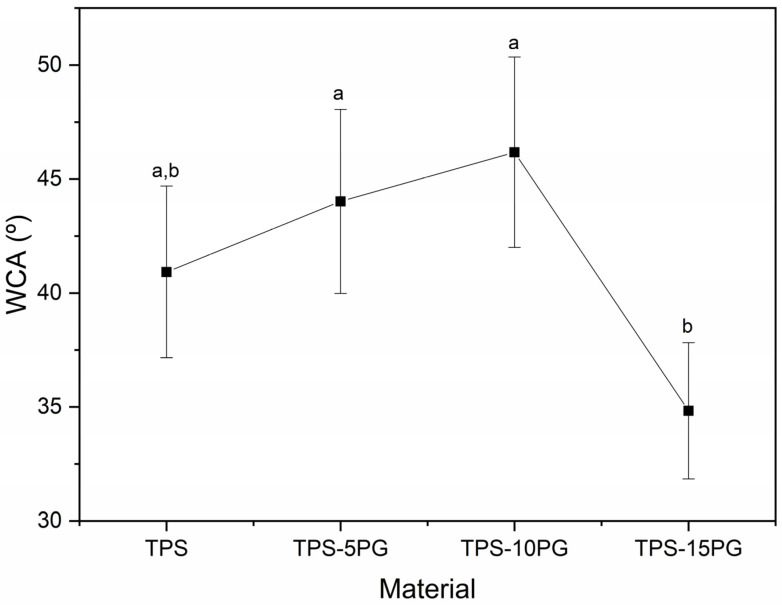
Water contact angle of thermoplastic starch (TPS) and formulations with 5, 10, and 15 phr of peach gum (PG). ^a,b^ Different letters within the same property show statistically significant differences between formulations (*p* < 0.05).

**Figure 8:**
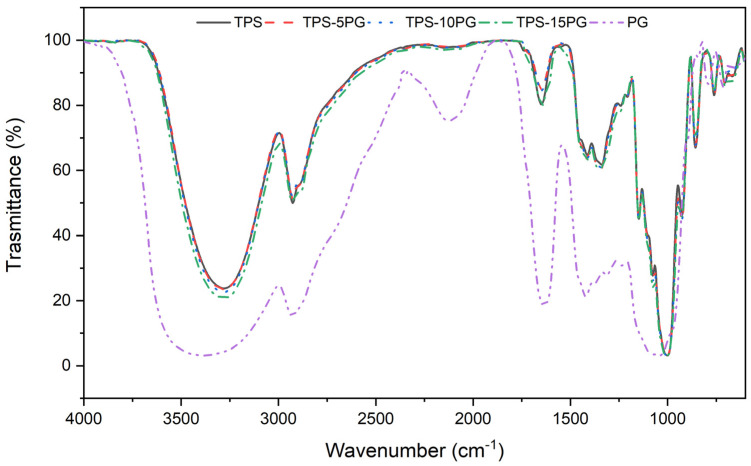
Fourier Transform Infrared Spectroscopy (FTIR) spectra of thermoplastic starch (TPS) and formulations with 5, 10, and 15 phr of peach gum (PG) and pure PG.

Figure 7 shows the variation in water contact angle with PG content. The plot shows that all the materials display a hydrophilic character as the WCA is lower than 65° [43]. It is observed that there are no significant differences (*p* > 0.05) in the contact angle of TPS with the addition of PG in 5 and 10 phr. However, when PG was added in 15 phr, the WCA value was statistically lower than TPS, TPS–5PG, and TPS–10PG. The contact angle highly depends on the surface’s topographical and chemical properties [44]. Therefore, as no chemical changes were detected in the formulations due to the addition of peach gum (Figure 8), the wettability of the samples could be related to the surface topography. TPS–5PG and TPS–10PG show excellent miscibility with TPS, as seen in Figure 6b,c; therefore, the materials may have a homogeneous surface. On the contrary, TPS–15PG presents a lack of miscibility between PG and TPS (Figure 6d), which could have caused superficial defects in the sample that may lead to a reduction in the WCA in this sample.

### 3.6. Water Sensitivity

The TPS and TPS–PG formulations’ water sensitivity regarding dissolved mass is shown in Table 3. Both pure TPS and raw PG present no significant differences in their water sensitivity (*p* > 0.05). However, it is observed that the solubility of TPS–PG formulations significantly increased with PG. A content of 5 phr of PG increased the water sensitivity by 6%, and a content of 10 and 15 phr of PG increased it by 24%.

It is important to note that even when TPS-based materials tend to be hydrophilic and hygroscopic, the TPS water solubility index is low [45]. Thus, the soluble fraction of the films is related to the amount of starch that dissolves in the water [45] and the plasticizers that migrate to the water during immersion, as reported by [46]. Therefore, PG did influence the water sensitivity of TPS, which two causes could explain. First, the presence of arabinogalactans in the composition of the TPS–PG formulations increased the water sensitivity because these polysaccharides are water-soluble [17]. Second, PG absorbs and retains water due to its swelling capacity, simultaneously increasing the TPS structure’s free volume. This increase in free volume favored water absorption due to a more open structure [46] increasing the contact area between TPS and water and enhancing the ability of the water to dissolve the remaining soluble starch.

TPS–10PG and TPS–15PG presented statistically the same water sensitivity, which suggests that the soluble starch in the formulations is the same and that its total solubility has been reached with a PG content of 10 phr.

### 3.7. Chemical Characterization

Figure 8 compares the FTIR spectra of pure TPS and TPS–PG formulations. The TPS FTIR spectrum shows the typical absorption bands corresponding to starch and glycerol functional groups. The peaks over 3270 cm^−1^ indicate hydrogen bond vibration and O-H stretching. A peak at 2930 cm^−1^ indicates an asymmetric stretching vibration typical of the C-H bond. The absorption peaks at 1651 cm^−1^ and 1150 cm^−1^ are produced by O-H bending vibration and C-O-C asymmetric stretching, respectively [47,48,49]. The raw PG spectrum presents bands associated with the composition of PG polysaccharides. The band at 3414 cm^−1^ corresponds to the O-H stretching vibration of the hydroxyl group. The bands at 2928 cm^−1^ and the shoulder at 2864 cm^−1^ are related to the C-H stretching vibrations. The high-intensity peak at 1626 cm^−1^ corresponds to COO-stretching derived from the glucuronic acid component, and the peak at 1066 cm^−1^ is attributed to the C-O stretching vibration [17,50]. Because TPS and PG are composed of polysaccharides and have the same characteristic groups, it is rather difficult to determine changes in the TPS–PG formulations’ spectra concerning the pure TPS spectrum.

**Figure 9:**
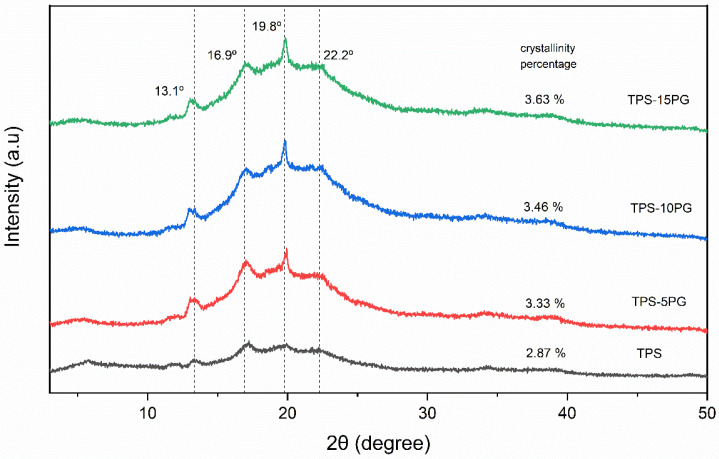
X-ray diffraction intensity scans for thermoplastic starch (TPS) and formulations with 5, 10, and 15 phr of peach gum (PG).

The X-ray diffractograms of TPS and the TPS–PG formulations are displayed in Figure 9. The X-ray diffractograms of all the materials show they exhibited the typical behavior of semicrystalline materials. Similar diffractograms were obtained in previous works for thermoplastic starch obtained from corn starch [8].

In the diffractograms, the characteristic peaks of TPS are observed at 2θ = 13.1°, 16.9°, 19.8°, and 22.2°. The peaks at 2θ = 16.9° and 22.2° are related to the A-type structure typical of cereal-based starches [51], and those at 2θ = 16.9° are associated with the interactions between TPS’s external amylopectin chains and glycerol during thermo-mechanical processing [51,52]. The peaks at 2θ = 13.1° and 19.8° are associated with glycerol–starch hydrogen bonding interactions and indicate a V_H_-type structure, while the broad hump centered on 19° is characteristic of TPS [52,53].

The crystallinity percentage of TPS is 2.87%, which could be related to the residual crystallinity of the remaining starch granules without plasticizing and to the parallel orientation of the starch chains, which leads to retrogradation (starch recrystallization) [54]. All the TPS–PG formulations present higher crystallinity than pure TPS, which increases as the PG content increases in the formulations. This is because of the intrinsic crystallinity of the polysaccharides in raw PG [19].

### 3.8. Disintegration under Composting Conditions

**Figure 10:**
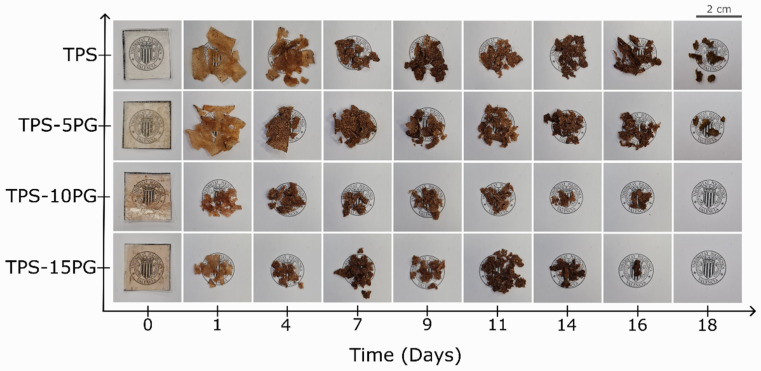
Visual appearance of thermoplastic starch (TPS) and formulations with 5, 10, and 15 phr of peach gum (PG) during the disintegration test in controlled compost conditions in terms of the elapsed time.

**Figure 11:**
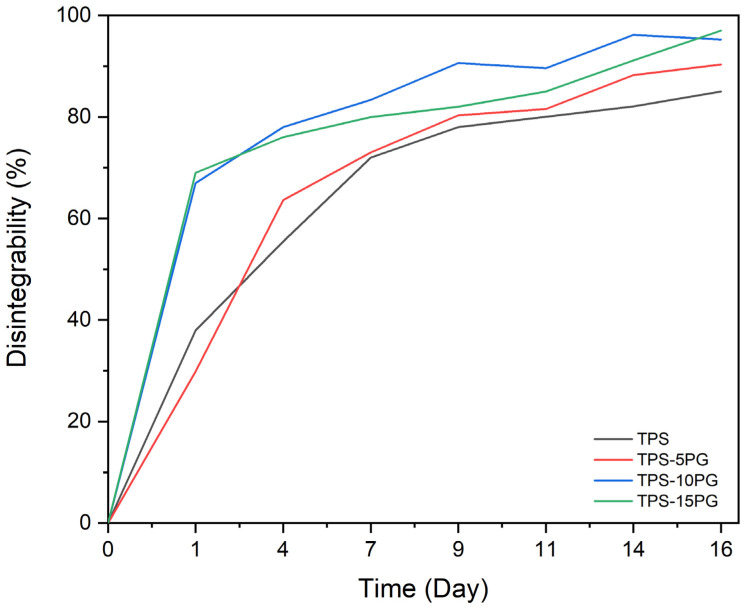
Disintegration degree of thermoplastic starch (TPS) and formulations with 5, 10, and 15 phr of peach gum (PG) under controlled compost conditions as a function of incubation time.

The biodegradable nature of TPS-based materials is one of the most striking qualities of this biomaterial [55]. Therefore, a disintegrability test was conducted under composting conditions at a laboratory scale to determine raw PG’s effect on TPS’s compostability. Figure 10 shows the visual appearance of TPS and TPS–PG formulations recovered at different composting times. Figure 11 presents the degree of disintegrability evaluated in terms of mass loss as a function of incubation time. Figure 10 shows that at day 0, all samples are transparent, which readily allows us to see clearly through them. After just one day in the composting reactor, all the samples lost their transparency and broke down. TPS and TPS–5PG lost around 38% of their weight, while TPS–10PG and TPS–15PG lost 70%. This transparency loss and breakage effects are linked to the beginning of the hydrolytic degradation of TPS and the formation of low-molecular-weight compounds. Hydrolytic degradation occurs due to hydroxyl or polar groups in the biopolymer [56]. This rapid beginning of degradation was previously reported for corn-starch-based TPS [8]. At day 7, the coloration of the sample turns darker, presumably due to microbial growth [57]. TPS and TPS–5PG lost 70% of their weight, while TPS–10PG and TPS–15PG lost 80%. On day 16, TPS–10PG and TPS–15PG were disintegrated, reaching 95% and 97% of weight loss. On day 18, TPS and TPS–5P reached 95–98% values, which are considered wholly degraded. The results show that thermoplastic starch is hydrolyzed and metabolized rapidly. The biodegradation of starch-based polymers is a result of an enzymatic attack at the glucosidic linkages between the sugar units, leading to the breakdown of long-chain sugar units into oligosaccharides, disaccharides, and monosaccharides that are readily accessible to microbial or enzymatic attack [55,56].

The visual appearance of the samples containing 10 and 15 phr of PG shows an increased disintegration in the early incubation period compared to pure TPS. This effect could be ascribed to an earlier hydrolytic reaction on the samples due to the increased water affinity as PG content increases in the formulations. The results are in correlation with the increased water sensibility percentage presented for these formulations.

## 4. Conclusions

Homogeneous TPS-based materials were prepared using rice starch (water and glycerol as plasticizers). The resulting TPS was then blended with peach gum through an extrusion process, followed by injection molding. The mechanical characterization results indicate that the TPS structure gradually turned stiffer due to retrogradation. The addition of 5 phr of PG did not significantly change TPS’s tensile behavior, and the addition of 15 phr of PG reduced the tensile properties of TPS. Moreover, TPS–10PG delayed the stiffening of the structure without altering TPS’s tensile properties. The electron scanning microscopy showed that PG added in 5 and 10 phr presented an excellent incorporation into the TPS matrix without phase separation.

Nonetheless, PG added in 15 phr produces a phase separation due to a lack of cohesion produced by low miscibility between PG and TPS, which explained the decrease in tensile properties. In addition, no significant differences were found in the WCA of TPS and TPS–PG formulations. However, a 24% water sensitivity increase was observed with a content of 10 and 15 phr of PG due to water-soluble polysaccharides in the composition. This increase in water sensitivity also increased the disintegration in the early stages of degradation, which corresponds to the hydrolytic degradation of the samples. Therefore, raw PG can be added as a sustainable additive to modify the biodegradation and water sensitivity of TPS, and at a content of 10 phr it helps to delay the retrogradation of the TPS structure, increasing its shelf life.

## Figures and Tables

**Table 1:** Composition of TPS–PG formulations.

		Resin Content (phr)		
Matrix	Resin	5	10	15
TPS	Peach gum	TPS–5PG	TPS–10PG	TPS–15PG

**Table 2:** CIEL*a*b* color coordinates of thermoplastic starch (TPS) and formulations with 5, 10, and 15 phr of peach gum (PG).

Title 1	L*	a*	b*	YI E313	ΔE*
TPS	38.91 ± 0.26 ^a^	−1.54 ± 0.05 ^a^	5.05 ± 0.05 ^a^	17.53 ± 0.08 ^a^	0 ^a^
TPS–5PG	42.96 ± 0.20 ^b^	0.08 ± 0.28 ^b^	8.18 ± 0.71 ^b^	29.03 ± 2.38 ^b^	5.37 ^b^
TPS–10PG	51.80 ± 1.34 ^c^	1.77 ± 0.39 ^c^	11.87 ± 1.32 ^c^	37.35 ± 3.33 ^c^	14.95 ^c^
TPS–15PG	43.56 ± 1.04 ^b^	−0.03 ± 0.07 ^b^	7.90 ± 0.25 ^b^	27.70 ± 0.83 ^b^	5.66 ^b^

^a–c^ Different numbers show statistically significant differences between the formulations (*p* < 0.05).

**Table 3:** Solubility of raw peach gum (PG), thermoplastic starch (TPS), and formulations with 5, 10, and 15 phr of peach gum (PG).

Formulation	Water Sensitivity (%)
PG	29.8 ± 1.2 ^a^
TPS	28.9 ± 1.0 ^a^
TPS–5PG	31.7 ± 0.8 ^b^
TPS–10PG	36.3 ± 1.2 ^c^
TPS–15PG	36.8 ± 0.8 ^c^

^a–c^ Different numbers show statistically significant differences between the formulations (*p* < 0.05).

## Data Availability

The data presented in this study are available on request from the corresponding author.
